# *Araceae* root and citrus fibers tend to decrease *Escherichia coli* adhesion and myeloperoxidase levels in weaned piglets

**DOI:** 10.3389/fvets.2023.1111639

**Published:** 2023-04-28

**Authors:** Sofie Tanghe, Maartje De Vos, Jeroen Degroote, Kobe Lannoo, Jan Vande Ginste, Romain D'Inca, Joris Michiels

**Affiliations:** ^1^Nutrition Sciences N.V., Drongen, Belgium; ^2^Laboratory for Animal Nutrition and Animal Product Quality, Department of Animal Sciences and Aquatic Ecology, Faculty of Bioscience Engineering, Ghent University, Ghent, Belgium

**Keywords:** weaning, piglet, gut health, immunity, fiber, citrus, *Araceae* root, *E. coli*

## Abstract

**Introduction:**

Weaning is a stressful experience in the piglet's life, and it often coincides with impaired gut health. Post-weaning diarrhea in piglets is frequently caused by enterotoxigenic *Escherichia coli* (*E. coli*). The first step of an *E. coli* infection is the adhesion to host-specific receptors present on enterocytes, leading to pro-inflammatory immune responses. The aim of this study was to examine if specific fiber fractions in the piglet diet can prevent *E. coli* adhesion and subsequent immune responses.

**Methods:**

The trial included 200 piglets (Danbred × Piétrain): 10 piglets/pen × 10 pens/dietary treatment × 2 dietary treatments. From weaning until 14 days (d14) post-weaning, piglets were fed a control diet or test diet with 2 kg/ton of a mixture of specific fiber fractions derived from *Araceae* root and citrus. Afterwards, 1 piglet per pen was euthanized, a section was taken at 75% of small intestinal length and *E. coli* colonization on the mucosal epithelium was quantified by scraping and conventional plating. From the same small intestinal section, histo-morphological indices were assessed, and mucosal scrapings were analyzed for gene expression of pro- and anti-inflammatory cytokines, and NF-kB. Analyses of specific intestinal bacteria and SCFA were performed on samples of intestinal content (small intestine, caecum, colon). Fecal samples were taken to measure myeloperoxidase (MPO), calprotectin and PAP/RAG3A as biomarkers for intestinal inflammation.

**Results and discussion:**

Piglets fed the fiber mixture tended to have decreased *E. coli* colonization to the mucosal epithelium (5.65 vs. 4.84 log10 CFU/g; *P* = 0.07), less *E. coli* in the caecum (8.91 vs. 7.72 log10 CFU/g; *P* = 0.03) and more Lachnospiraceae in the colon (11.3 vs. 11.6 log10 CFU/g; *P* = 0.03). Additionally, the fiber mixture tended to increase cecal butyric acid (10.4 vs. 19.1 mmol/kg; *P* = 0.07). No significant effect on histo-morphological indices and on gene expression of pro- and anti-inflammatory cytokines and NF-kB was observed. The fecal MPO concentration tended to decrease (20.2 vs. 10.4 ng/g; *P* = 0.07), indicating less intestinal inflammation. In conclusion, this study showed that specific fiber fractions from *Araceae* root and citrus in piglet weaner diets may decrease the risk of pathogen overgrowth by reducing *E. coli* adhesion and intestinal inflammation.

## 1. Introduction

Enterotoxigenic *Escherichia coli* (ETEC) F4 and F18 are an important cause of diarrhea in neonatal and recently weaned piglets, leading to substantial economic losses due to reduced growth rate, cost of medication and mortality (1). Nguyen et al. (1) showed that the chance to find F4 or F18 *E. coli* genetically susceptible pigs in the Flemish pig population is 98%, indicating a high risk for piglets to develop post-weaning diarrhea due to ETEC infections.

The first step of an *E. coli* infection is the adhesion to host-specific receptors present on the enterocytes of the small intestine. This adhesion to the intestinal glycoproteins is mediated through adhesins which are expressed in the ETEC fimbriae, and which are different between ETEC F4 and F18. The ability of ETEC for this adhesion to the small intestine is a crucial factor in the establishment of diarrhea, as it allows ETEC to overcome the flushing action of small intestinal peristalsis and to colonize the intestinal epithelium. Once ETEC has adhered and colonized the small intestine, it can produce enterotoxins, which cause electrolyte and fluid losses, leading to acute and severe aqueous diarrhea (2).

Pathogens in the intestine are recognized in the mucosal immune system by pattern recognition receptors (PRR), which recognize bacterial conserved patterns. After recognition, various signaling cascades are activated, which lead to the production of immune-activating and immune-regulatory cytokines (3). Toll-like receptors (TLR) are the best studied family of PRR. They recognize certain patterns on pathogenic bacteria, known as pathogen associated molecular patterns (PAMP). Well-known PAMP are lipopolysaccharides (LPS), which are endotoxins located in the outer membrane of Gram negative bacteria, such as *E. coli*. Lipopolysaccharides are released during active cellular growth and after bacterial cell lysis, initiating local and even systemic inflammations. Toll-like receptor 4 is regarded as the major PRR for LPS sensing. The stimulation of TLR4 by LPS activates cells through eliciting the NF-kB signaling pathway, which leads to a higher production of pro-inflammatory cytokines that are necessary to activate immune responses (4). Furthermore, Xiao et al. (5) have shown that LPS stimulation in newly weaned piglets not only leads to a release of proinflammatory cytokines, but it can also result in profound changes in the gut microbial composition, probably caused by the interplay with the immune response, and in histological erosion of the ileum, implying a lower surface area for nutrient absorption and a detrimental effect on gut health.

Dietary fibers are known for their beneficial effects on gut health and gastrointestinal immunity. They can act as prebiotics and promote the growth of beneficial bacteria in the gut. Dietary fibers can also be a fermentation substrate for commensal bacteria and enhance the production of short chain fatty acids (SCFA), which play several roles in metabolism and immunity. G-protein coupled receptors can bind SCFA at the mucosa, mediating the regulatory action of SCFA on inflammatory processes (3). Furthermore, dietary fibers can inhibit the growth of pathogens by preventing their adhesion to epithelial cells. Fibers, such as human milk oligosaccharides and pectins can serve as decoy receptors, thereby allowing pathogens to adhere to dietary fibers, and not to epithelial cell receptors, leading to displacement from the gastrointestinal tract (6). Mannose-directed adhesion is a very common described binding ability of bacteria and hence pathogen adhesion can be reduced by mannose or mannan containing compounds (7, 8).

Next to these indirect effects of dietary fibers on the immune system, dietary fibers might also have a direct immunomodulatory effect. Dietary fibers such as pectins can strengthen the mucus layer and gut barrier function by stimulating mucin secretion of goblet cells or by mucoadhesive properties, thereby limiting the passage of harmful substances and preventing activation of inflammatory responses (9). Dietary fiber can also be recognized by PRR on epithelial and immune cells in the intestine. Toll-like receptors have demonstrated to be involved in the recognition of dietary fibers, and specific fiber fractions can have the capacity to bind TLR (10–13). Pectin is one of the dietary fibers with TLR binding capacity and possible anti-inflammatory effects. Various mechanisms through which pectins may affect TLR-mediated immune modulation can be proposed. Vogt et al. (11) postulated that pectins could bind to TLR4 and cause competitive inhibition of LPS-mediated activation. Chen et al. (14) observed that pectins could bind to LPS and in this way decrease the binding of LPS to TLR4. Sahasrabudhe et al. (12) and Beukema et al. (13) demonstrated a true blocking of TLR receptors by pectins, and Sahasrabudhe et al. (12) showed that the mechanism of action by which pectins could inhibit TLR2-1 was via direct binding to the TLR2 ectodomain through electrostatic forces. In case of persistent inflammation, this blocking or suppressed activation of TLR by pectins can be of interest as it will limit the overstimulation of TLR, leading to a controlled inflammatory response and reduced inflammation (12). Dietary fibers such as pectins and fructans can also have a direct protective effect on the epithelial barrier function by binding to TLR2, which serves as an important barrier regulatory receptor (11, 15, 16).

The objective of this study was to examine if specific fiber fractions derived from *Araceae* root and citrus in the piglet diet can prevent *E. coli* adhesion and intestinal inflammation. We hypothesized that the *Araceae* root fiber fraction, which is rich in mannans, could prevent *E. coli* adhesion to the receptors, and that the citrus fiber fraction, which is rich in pectins, could reduce inflammation through the above mentioned effects on microbiota composition, SCFA production, mucus strengthening or TLR binding. Therefore, an *in vivo* trial was set up where weaned piglets were fed a diet with or without specific fiber fractions derived from *Araceae* root and citrus, and *E. coli* colonization of the mucosal epithelium, histo-morphological indices, gene expression of pro- and anti-inflammatory cytokines and NF-κB, specific intestinal bacteria, SCFA, and biomarkers for intestinal inflammation were determined.

## 2. Material and methods

### 2.1. Animals and diets

The study was performed according to the ethical standards and recommendations for accommodation and care of laboratory animals covered by the European Directive 2010/63/EU on the protection of animals used for scientific purposes and the Belgian royal decree KB29.05.13 on the use of animals for experimental studies. No licensed ethical approval was required as this study did not involve procedures causing harm equivalent to, or higher than, that caused by the introduction of a needle in accordance with good veterinary practice, and because animals were killed solely for the use of their organs or tissues (2010/63/EU).

The experiment was carried out at a commercial pig farm in Bissegem, Belgium. The farm had a 220 head sow herd, operating in a 4-week batch system with weaning on 23 days of age. Breeds used were Danbred sows and Belgian Piétrain boars. The experiment included non-castrated males and females. Ten piglets were housed per pen of 3.0 m^2^ with plastic slatted floors, plastic fences and polyester concrete floor heating system. Pens were equipped with a single-sided stainless steel dry feeder (60 cm wide, 3 eating spaces) and a bowl drinker. Temperature and ventilation rate during the experiment were computer-controlled and appropriate for the piglet age. Ambient temperature was set at 30 °C and a 24 L schedule until d5 post-weaning, and thereafter it was linearly adjusted to 26 °C with a 18L:6D light schedule.

The experiment included 2 dietary treatments, with 10 replicates (pens) per treatment and 10 piglets per pen. The experiment was replicated over time and divided in 2 consecutive rounds with a 4 week time difference. The trial included 200 piglets in total: 10 piglets/pen × 5 pens/round/dietary treatment × 2 rounds × 2 dietary treatments.

For each round, the following experimental setup was followed. At weaning (23 ± 0.72 days of age), piglets were selected for sex and median body weight. Piglets showing visuals signs of clinical illness, lower vitality, arthritis and umbilical or inguinal hernia were excluded from the selection. Piglets were allocated to the pens based on the following criteria in the specified order: 1) pens either hold male or female piglets, 2) 0.10 kg tolerance on the average starting body weight between pens and 3) 0.20 kg tolerance on the starting body weight standard deviation within a pen between pens. Treatments were then allocated to the pens in order to stratify for average starting body weight (tolerance of 0.02 kg), and having a similar sex distribution between treatments within each round (20% tolerance). Litter origin and age were not included as a selection or allocation criterium and were treated randomly.

Two dietary treatments were compared in this trial: the control diet (Control) and the test diet (Test) supplemented with 2 kg/ton of specific fiber fractions derived from *Araceae* root and citrus (Vitafibra, Nuscience, Belgium). The diet was produced in one batch and split in two equal parts. One part served as the Control diet. To the other part, 2 kg/ton of specific fiber fractions derived from *Araceae* root and citrus was added and it was thoroughly mixed again to ensure homogeneity of the Test diet. The composition of the diets is given in Table 1. Piglets were fed the experimental diets from d0 to d14 post-weaning. All diets were fed *ad libitum*.

**Table 1:** Diet composition.

**Ingredients (%)**	**Control**	**Test**
Barley	28.0	27.9
Wheat	20.0	20.0
Corn heat treated	13.1	13.1
Full fat soya beans	10.0	10.0
Soya protein concentrate	5.5	5.5
Wheat heat treated	5.0	5.0
Oatmeal	2.5	2.5
Soya bean meal	2.5	2.5
Soya bean oil	1.3	1.3
L-Lysine HCl	0.52	0.52
L-Threonine	0.22	0.22
DL-Methionine	0.18	0.18
L-Tryptophan	0.06	0.06
L-Valine	0.10	0.10
Salt	0.46	0.46
Monocalcium phosphate	0.26	0.26
Limestone	0.26	0.26
Concentrate^a^	10.0	10.0
Fiber mixture^b^		0.2
**Calculated nutrient composition**		
NE (kcal/kg)	2500	2497
Dry matter (%)	89.7	89.7
Crude protein (%)	16.5	16.5
Crude fat (%)	6.1	6.1
Crude ash (%)	4.1	4.1
Crude fiber (%)	4.0	4.0
Starch ewers (%)	40.4	40.3
Sugars (%)	7.5	7.5
Lactose (%)	4	4
Calcium (%)	0.4	0.4
Phosphorus (%)	0.4	0.4
Sodium (%)	0.2	0.2
SID Lys (%)	1.05	1.05
SID Met + Cys (%)	0.62	0.62
SID Thr (%)	0.69	0.69
SID Trp (%)	0.22	0.22
SID Val (%)	0.73	0.73

^a^The concentrate contained dairy products, vitamins, trace elements, flavoring compounds, enzymes, organic acids and MCFA, providing the following quantities per kg of diet: vitamin A, 15000 IU; vitamin D3, 2000 IU; vitamin E, 100 mg; vitamin K3, 3 mg; vitamin B1, 1.5 mg; vitamin B2, 5.4 mg; calcium D-pantothenate, 17.9 mg; vitamin B6, 2.9 mg; vitamin B12, 0.04 mg; nicotinamide, 30.3 mg; choline chloride, 500 mg; biotin, 0.1 mg; betaine, 100 mg; iron(II) sulfate monohydrate, 80 mg; ferrous chelate of glycine hydrate, 40 mg; cupric chelate of glycine hydrate, 140 mg; zinc sulfate monohydrate, 50 mg; zinc chelate of glycine hydrate, 50 mg; manganese(II) oxide, 80 mg; calcium iodate, 1 mg; sodium selenite, 0.30 mg; zinc-L-selenomethionine, 0.05 mg; endo-1,3(4)-beta-glucanase, 900 BGU; endo-1,4-beta-xylanase, 4000 XU; 6-phytase, 750 FTU. ^b^Specific fiber fractions derived from *Araceae* root and citrus (Vitafibra, Nuscience, Belgium), with the following composition: 380 g/kg soluble dietary fiber; 130 g/kg insoluble dietary fiber; 510 g/kg total dietary fiber; 138 g/kg mannose; 57 g/kg polygalacturonic acid.

### 2.2. Experimental and analytical procedures

#### 2.2.1. Piglet performance and health

Individual piglet body weight was registered on d0 and d14 post-weaning. Feed intake per pen was measured periodically (at d0 and d14) by weighing the feeders and the amount of feed offered. The average daily feed intake was then calculated by subtracting the feed rest from the amount of feed offered and taking into account the feed access days for each animal in the pen. Average body weight, average daily gain and feed conversion ratio (FCR) were calculated for each pen.

Monitoring of the piglets for any abnormalities, such as abnormal behavior and clinical signs of sickness, occurred daily. Piglets were individually medicated by intramuscular injections only if necessary in case of poor health. All deviations from normal and required medical treatments were recorded. If piglets were removed from the experiment because of death or poor health, the piglet weight and the date and reason for removal was recorded, based on judgement of the farm staff.

A visual assessment of the fecal consistency score of each pen was performed daily at 17h00 throughout the 14 days post-weaning period. An ordinal scoring system consisting of 4 categories was used: score 1 = firm and shaped, score 2 = soft and shaped, score 3 = loose, and score 4 = watery. Scores 1 and 2 were considered normal, and scores 3 and 4 were judged as diarrhea. Multiple fresh fecal droppings were evaluated for each run, and the daily maximum score for each pen was retained as the result from the observation. The diarrhea incidence was simultaneously assessed by counting the number of piglets with diarrhea when a pen received a fecal consistency score 3 or 4. Here, piglets were individually inspected (visually, from a distance) for clear signs of diarrhea: filthy, wet backside and tail, dehydrated, loss of condition, and irritation of the skin around the anus.

#### 2.2.2. Histo-morphological indices

At d14, one piglet per pen, having the median body weight of that pen, was selected for the collection of gastro-intestinal tissues and digesta. Animals were first sedated by electronarcosis, followed by euthanasia by exsanguination. The abdominal cavity was opened and the entire gastro-intestinal system was removed. The small intestine was isolated and spread to its full length. Subsequently, a section at 75% of small intestinal length (measured from the side of the pylorus) was taken, rinsed with saline (0.9% NaCl), and fixated in a 4% formaldehyde solution. These samples were used to assess histo-morphological indices, i.e., villus length, villus width, crypt depth, intra-epithelial lymphocytes and goblet cells according to Van Nevel et al. (17) and Van Noten et al. (18). Per piglet, the number of villi with adjacent crypts suitable for measurement varied between 9 and 16. The villus/crypt ratio and the villus surface area were calculated, the latter based on the formula described by De Vos et al. (19).

#### 2.2.3. *E. coli* colonization of the mucosal epithelium

From the same small intestinal section at 75% of length, colonization by *E. coli* on the surface of the mucosal epithelium was quantified by rinsing a 5 cm section with saline, scraping of the mucosa with a glass slide and conventional plating (17). In brief, bacterial counts (viable counts; log10 colony-forming units (CFU) /g mucosa) were obtained using the ring-plate technique (20). Seven serial 10-fold dilutions were made from 1 g of fresh mucosa in a sterilized peptone solution (peptone, 1 g/L; agar, 0.4 g/L; NaCl, 8.5 g/L and cysteine, 0.7 g/L) and plated onto selective media for counting *E. coli* (Tryptone Bile X-Glucuronide Agar, CM0945B, Oxoid; incubated for 24h at 37°C, aerobically). Data were log10 transformed prior to statistical testing. The lower limit of detection was 2 log10 CFU/g.

#### 2.2.4. Gene expression

Mucosal scrapings at 75% small intestinal length were also analyzed for gene expression by reversed transcribed real-time qPCR, for the following genes: *MUC2, IL1*β, *IL6, TNF*α, *IL10, IFNG, TLR4, NF*κ*B1, RELA* (NF-κB p65), *OCLN*, and *TJP1*. In brief, mucosal total RNA was extracted using the Bio-Rad Aurum Total RNA Fatty and Fibrous Tissue Kit (Bio-Rad Laboratories, Inc., Hercules, USA) according to the manufacturer's instructions, including an on-column DNase I treatment to remove genomic DNA (gDNA). The concentration and purity (OD260/280) of RNA were determined with the NanoDrop ND-1000 (NanoDrop Technologies, Thermo Scientific, Wilmington, USA). One μg RNA was analyzed by 1% agarose gel electrophoresis to check RNA integrity (28S and 18S rRNA bands). Furthermore, a minus-RT control PCR was performed using *YWHAZ* as primer to verify the absence of any gDNA contamination. Afterwards, 1 μg of high-quality DNA-free RNA was reverse transcribed in the 20 μL reverse-transcription reaction with the ImProm-II cDNA synthesis kit (Promega, Madison, USA), containing both oligo dT and random primers. The obtained cDNA was diluted 10 times with molecular grade water and a control PCR using 2 μL cDNA was performed to verify the reverse-transcription reaction. Primers used for genes in the study were designed with Primer3Plus (Table 2). The repeats, the secondary structure and single nucleotide polymorphism in the target sequence were checked with RepeatMarker, mfold and dbSNP, respectively. All these primer sequences were gene isoform specific as they were designed based on certain exon-exon boundaries of published pig gene sequences corresponding to the accession number. Primers were then purchased from IDT (Integrated DNA Technologies, Leuven, Belgium). The RT-qPCR was carried out on the CFX96 Touch Real-Time PCR Detection System (Bio-Rad Laboratories, Inc.). Two μL cDNA template, 5 μL 2X KAPA SYBR FAST qPCR Kit Master Mix (Kapa Biosystems, Inc., Wilmington, USA), 2 μL molecular grade water, 0.5 μL forward primer and 0.5 μL reverse primer (5 μmol/L each) were added to a total volume of 10 μL. The amplification conditions were as follows: (1) enzyme activation and initial denaturation (95°C for 3 min); (2) denaturation (95°C for 20 s) and annealing/extension and data acquisition (annealing temperature depending on primer for 40 s) repeated 40 cycles; and (3) dissociation (melt curve analysis from 70 to 90°C with 0.5°C increment every 5 s). The primers used in this study were first optimized by gradient quantitative real-time PCR. A 5-fold dilution series (5 points, from 1 to 625 times dilution) of cDNA as a standard curve was included at 3 gradient temperatures to determine PCR amplification efficiency and specificity. The standard curve was also included in each run to determine PCR efficiency. In this study, PCR amplification efficiencies were consistently between 90 and 110%. Gene-specific amplification was verified by agarose gel electrophoresis and melting curve analysis. Efficiency was used to convert the Cq value into raw data. Data were normalized to the geometric mean of reference genes *HPRT* (M value 0.44) and *YWHAZ* (0.44) and scaled to control treatment according to Hellemans et al. (21).

**Table 2:** Primer sequences used for reverse-transcription quantitative real-time PCR.

**Gene symbol^a^**	**Accession number**	**Primer**		**Product length (bp)**	**Ta (°C)**
*MUC2*	NM_002457.4	Reverse	CATGCTGGAGCTGGACACTA	97	60
		Forward	GCCGTCAGAGAGGAATTCTG		60
*IL1B*	NM_214055.1	Reverse	AGTGGAGAAGCCGATGAAGA	113	60
		Forward	CATTGCACGTTTCAAGGATG		60
*IL6*	NM_214399.1	Reverse	CCTCTCCGGACAAAACTGAA	118	60
		Forward	TCTGCCAGTACCTCCTTGCT		60
*TNF*	NM_214022.1	Reverse	CCCCCAGAAGGAAGAGTTTC	92	60
		Forward	CGGGCTTATCTGAGGTTTGA		60
*IL10*	NM_214041.1	Reverse	GTAATGCCGAAGGCAGAGAG	118	60
		Forward	CACAGGGCAGAAATTGATGA		60
*IFNG*	NM_213948.1	Reverse	CAAAGATAACCAGGCCATTCA	90	60
		Forward	TTCAGTTTCCCAGAGCTACCA		60
*TLR4*	NM_001113039.2	Reverse	TGTGCTGAGTTTCAGGAACG	87	60
		Forward	GCCACAGATACCAGGAGGAA		60
*NFKB1*	NM_001048232.1	Reverse	CTCGCACAAGGAGACATGAA	97	60
		Forward	GGGTAGCCCAGTTTTTGTCA		60
*RELA*	NM_001114281.1	Reverse	GGGGACTACGACCTGAATGC	167	60
		Forward	CTCCCCGAGTTCCGATTCAC		60
*OCLN*	NM_001163647.2	Reverse	TTCATTGCTGCATTGGTGAT	113	60
		Forward	ACCATCACACCCAGGATAGC		60
*TJP1*	XM_021098856.1	Reverse	GCGCTGACTTTTGGAGATTC	105	60
		Forward	ATGCAGGAAACTTGGTTTGG		60
*HPRT1*	NM_001032376.2	Reverse	GGCAAAACAATGCAAACCTT	98	60
		Forward	ACACTTCGAGGGGTCCTTTT		60
*YWHAZ*	NM_001315726.1	Reverse	AGGAGCCCGTAGGTCATCTT	89	60
		Forward	ATTCTCGAGCCATCTGCTGT		60

^a^*MUC2*: mucin 2; *IL1B*: interleukin 1 beta; *IL6*: interleukin 6; *TNF*: tumor necrosis factor; *IL10*: interleukin 10; *IFNG*: interferon gamma; *TLR4*: toll-like receptor-4; *NFKB1*: nuclear factor NF-kappa B 1; *RELA*: nuclear factor NF-kappa B p65 subunit; *OCLN*: occludin; *TJP1*: tight junction protein 1; *HPRT1*: hypoxanthine phosphoribosyltransferase 1; *YWHAZ*: tyrosine 3-monooxygenase/tryptophane 5-monooxygenase activation protein zeta.

#### 2.2.5. Sample collection with BioFreeze™ preservation solution

Samples of feces and intestinal content [distal small intestine (last 25%), caecum and mid-colon] were taken and immediately preserved using BioFreeze™ sampling kits (Alimetrics Diagnostics Ltd., Espoo, Finland), according to the manufacturer's instructions. A sample vial was used for initial digesta collection and homogenization. From the homogenized digesta, one evenly filled spoonful of sample, which equals approximately 1 g, was transferred into a BioFreeze™ vial containing 9 ml of BioFreeze™ preservation buffer. The spoon with the sample was placed into the vial, the cap was closed carefully, and the vial was shaken vigorously to completely suspend the sample material into the preservation buffer. After collecting all samples, the vials were placed back into the original delivery box and shipped to Alimetrics Diagnostics Ltd laboratory.

#### 2.2.6. Specific intestinal bacteria

The bacterial DNA was extracted from the samples for qPCR analysis according to the following protocol. A BioFreeze™ vial with intestinal content [distal small intestine (last 25%), caecum and mid-colon] was shaken vigorously so that the sample material was evenly mixed with the preservation buffer. Next, 2 mL of the suspension was transferred into a clean microcentrifuge tube and centrifuged at 17 000 × g for 10 min. Afterwards, the pellet was re-suspended in 600 μL of lysis buffer containing EDTA and Tris HCl with 20 μL of proteinase K, and the suspension was transferred to a screw-cap microcentrifuge tube containing 0.4 g of sterile glass beads. Then, the suspensions were incubated at 65°C for 60 min with vortexing for 30 s (1400 rpm) at 10-min intervals.

The bacterial cells were disrupted by two 1-min rounds of bead beating (MP Biomedicals, USA) at 6.5 m/s, after which genomic DNA was purified from the homogenates by adding 750 μL phenol-chloroform-isoamyl alcohol (25:24:1) and centrifuging at 10,000 × g for 10 min. Of the aqueous phase, 550 μL was collected, 600 μL of chloroform-isoamyl alcohol (24:1) was added and the mixture was centrifuged at 10,000 × g for 10 min. Of the aqueous phase, 350 μL was collected and DNA was precipitated by adding 0.6 volumes of 100% isopropanol and 35 μL of Na-acetate and pelleted by centrifugation at 18,000 × g for 10 min. Finally, the DNA pellet was washed twice with 1 mL of ice cold 70% ethanol, dried and re-suspended in 100 μL of Tris-EDTA buffer.

Selected abundant bacterial taxa present in upper and lower intestinal tract were quantified with 16S rRNA gene-targeted qPCR microbial assay using SYBR Green I chemistry, with the ProxiMap™ and DistaMap™ analysis panel, respectively (Alimetrics Diagnostics Ltd, Espoo, Finland).

#### 2.2.7. SCFA

A BioFreeze™ vial with intestinal content [distal small intestine (last 25%), caecum and mid-colon] was shaken vigorously and centrifuged as described in section 2.2.6. The formed supernatant was used as the SCFA sample. The SCFA profiles were analyzed by gas chromatography (Agilent Technologies, Santa Clara, CA, USA) with pivalic acid (Sigma-Aldrich, St. Louis, MO, USA) as an internal standard. The chromatography procedure used a glass column packed with 80/120 Carbopack B-DA/4% Carbowax stationary phase, helium as a carrier gas, and a flame ionization detector, and it has been described previously by Apajalahti et al. (22).

#### 2.2.8. Fecal biomarkers of intestinal inflammation

Pig Myeloperoxidase (MPO), Pig Regenerating Islet Derived Protein 3 Alpha REG3a (PAP/REG3a) and Pig Calprotectin were analyzed as fecal biomarkers of intestinal inflammation. A BioFreeze™ vial with fecal content was shaken vigorously and centrifuged as described in Section 2.2.6. The formed supernatant was diluted to meet the MPO, PAP/REG3a and Calprotectin kit providers detection range of the analyzed parameters. Pig MPO and Pig PAP/REG3a were analyzed using kits produced by Abbexa (abx154980 and abx361597, respectively). Both kits were based on sandwich enzyme-linked immunosorbent assays. Pig Calprotectin was analyzed using a kit produced by Cusabio (CSB-EQ013485PI), based on competitive inhibition enzyme immunoassay technology. Manufacturer's instructions were followed to perform the analysis.

### 2.3. Statistical analysis

Data were analyzed by linear mixed model analysis, with treatment as fixed effect and round as random effect, using the lme4 package and lmer function of the statistical software of R (23). As the assumptions of normality, independence and homogeneity were not met for the treatment incidence, this parameter was analyzed by generalized linear mixed model analysis, using the lme4 package and glmer function. Pen was considered as the experimental unit. Differences were considered significant at *P* < 0.05 and trends at *P* < 0.1.

## 3. Results

### 3.1. Piglet performance and health

No significant differences were observed between the two dietary treatments on piglet body weight, average daily gain, feed intake and feed conversion ratio (Table 3).

**Table 3:** Performance of piglets fed a control diet (Control; *n* = 10) or a diet containing 2 kg/ton specific fiber fractions derived from *Araceae* root and citrus (Test; *n* = 10) from d0-d14 post-weaning.

	**Control**	**Test**	**SEM**	***P*** **-value**
Body weight d0 (kg)	6.82	6.83	0.48	0.70
Body weight d14 (kg)	8.10	8.20	0.70	0.55
Average daily gain d0-d14 (g/day)	90.0	98.0	16.6	0.53
Feed intake d0-d14 (g/day)	163.8	167.2	24.2	0.78
FCR d0-d14	2.12	1.77	0.25	0.33

No piglet mortality was recorded during the trial. One piglet from the control diet was removed from the trial at day 10 for poor health. The animal was repetitively treated (Vetrimoxin Long Acting^®^ 150 mg/mL) during the last 7 days prior to removal for lower vitality, anorexia, swollen joints and rough hair coat, but did not respond to the treatment.

The piglets fed the test diet with the fiber mixture tended to have a better fecal consistency score during the first 14 days post-weaning (Table 4). No differences in diarrhea or antibiotic treatment incidences were observed between both dietary treatments.

**Table 4:** Fecal consistency, diarrhea incidence and antibiotic treatment incidence of piglets fed a control diet (Control; *n* = 10) or a diet containing 2 kg/ton specific fiber fractions derived from *Araceae* root and citrus (Test; *n* = 10) from d0-d14 post-weaning.

	**Control**	**Test**	**SEM**	***P*** **-value**
Fecal consistency score ^*^	1.90	1.71	0.07	0.07
Diarrhea incidence (%) ^**^	4.21	4.50	0.80	0.80
Treatment incidence (%) ^***^	0.34	0.30	0.31	0.80

* Fecal consistency was evaluated on a 4 point scale with score 1 = firm and shaped, score 2 = soft and shaped, score 3 = loose, and score 4 = watery. Scores 1 and 2 were considered normal, while scores 3 and 4 were considered as diarrhea.

** Diarrhea incidence was evaluated by daily counts of the piglets with diarrhea. Result express the average % of piglets that shows diarrhea per day.

*** Treatment incidence expresses the probability that a piglet is medically treated per day.

### 3.2. Histo-morphological indices

No significant differences were observed between the two dietary treatments on the crypt depth, villus length, villus width, villus surface area or villus/crypt ratio (Table 5). Also the number of intra-epithelial lymphocytes and goblet cells were not significantly different between the two dietary treatments (Table 5).

**Table 5:** Histo-morphological indices of the small intestine (section at 75% of small intestine length) of piglets at d14 post-weaning, when fed a control diet (Control; *n* = 10) or a diet containing 2 kg/ton specific fiber fractions derived from *Araceae* root and citrus (Test; *n* = 10) from d0 to d14 post-weaning.

	**Control**	**Test**	**SEM**	**P-value**
Crypt depth (μm)	299	291	12	0.63
Villus length (μm)	389	406	11	0.29
Villus width (μm)	112	116	4	0.42
Villus surface area (mm^2^)	0.136	0.150	0.007	0.13
Villus/Crypt	1.34	1.42	0.04	0.18
Intra-epithelial lymphocytes (count per 100 μm villus)	8.27	8.95	0.79	0.48
Goblet cells (count per 100 μm crypt)	7.21	8.98	1.28	0.28
Goblet cells (count per 100 μm villus)	2.10	2.70	0.39	0.19

### 3.3. *E. coli* colonization of the mucosal epithelium

**Figure 1:**
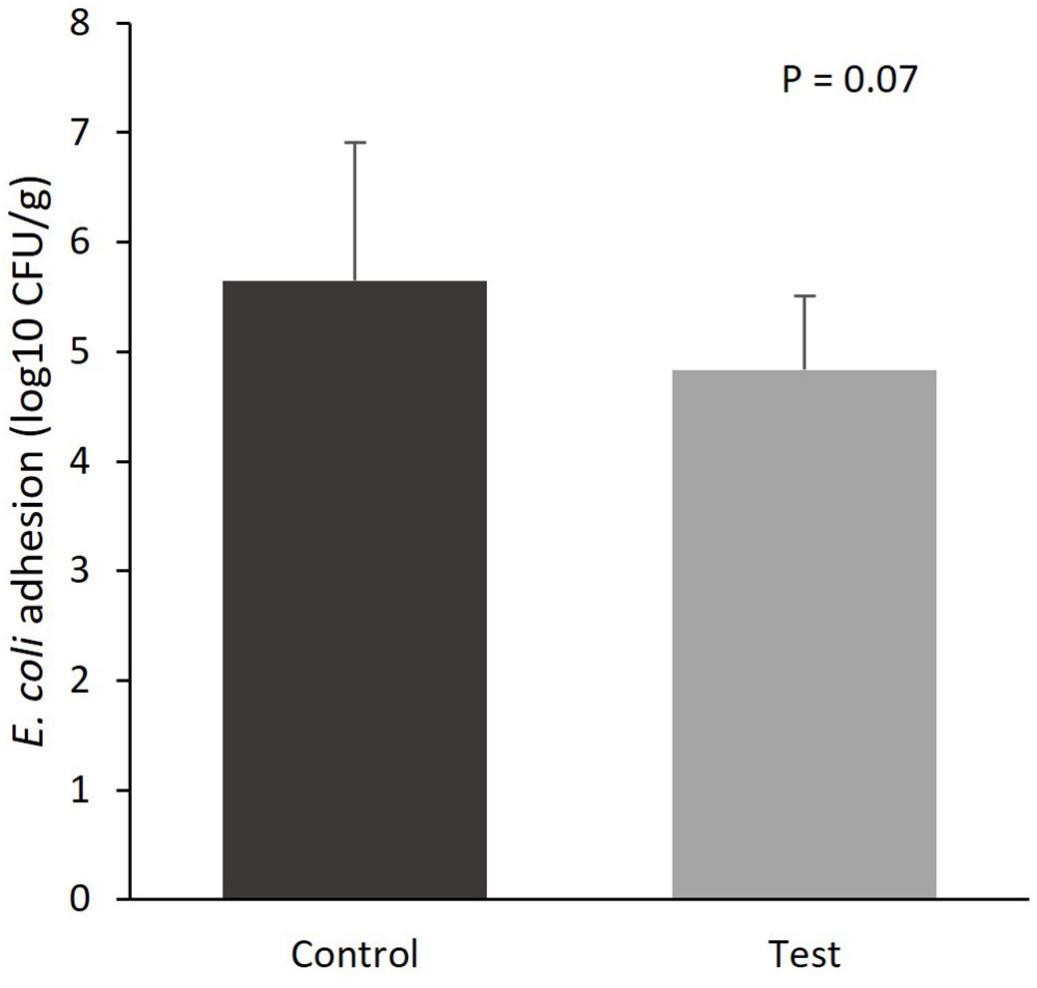
*E. coli* colonization of the mucosal epithelium of the small intestine (section at 75% of small intestine length) of piglets at d14 post-weaning, when fed a control diet (Control; *n* = 10) or a diet containing 2 kg/ton specific fiber fractions derived from *Araceae* root and citrus (Test; *n* = 10) from d0 to d14 post-weaning.

The colonization of *E. coli* to the mucosal epithelium of the small intestine tended to be lower for the piglets fed the diet containing specific fiber fractions derived from *Araceae* root and citrus (Figure 1; 5.65 vs. 4.84 log10 CFU/g, control vs. test diet, respectively; *P* = 0.07).

### 3.4. Gene expression

Gene expression in the mucosal epithelium of the small intestine was measured for genes related to mucus production (*MUC2*), inflammation (*IL1*β, *IL6, TNF*α, *IL10, IFNG, TLR4, NF*κ*B1* and *RELA*) and tight junctions (*OCLN* and *TJP1*). No significant differences in gene expression were observed between the two dietary treatments (Table 6).

**Table 6:** Gene expression in the mucosal epithelium of the small intestine (section at 75% of small intestine length) of piglets at d14 post-weaning, when fed a control diet (Control; *n* = 10) or a diet containing 2 kg/ton specific fiber fractions derived from *Araceae* root and citrus (Test; *n* = 10) from d0 to d14 post-weaning. Expressed relatively to the reference genes *HPRT1* and *YWHAZ*^a^.

	**Control**	**Test**	**SEM**	***P*** **-value**
*MUC2*	1.08	1.01	0.13	0.69
*IL1β*	1.32	0.98	0.32	0.39
*IL6*	1.42	0.83	0.29	0.20
*TNFα*	1.26	1.21	0.21	0.86
*IL10*	1.13	1.41	0.20	0.33
*IFNG*	1.29	1.40	0.27	0.77
*TLR4*	1.25	1.12	0.19	0.63
*NF-κB1*	1.10	0.89	0.19	0.33
*RELA*	1.14	0.86	0.31	0.14
*OCLN*	1.09	1.04	0.12	0.75
*TJP1*	1.22	1.12	0.51	0.68

^a^*MUC2*: mucin 2; *IL1B*: interleukin 1 beta; *IL6*: interleukin 6; *TNF*: tumor necrosis factor; *IL10*: interleukin 10; *IFNG*: interferon gamma; *TLR4*: toll-like receptor-4; *NFKB1*: nuclear factor NF-kappa B 1; *RELA*: nuclear factor NF-kappa B p65 subunit; *OCLN*: occludin; *TJP1*: tight junction protein 1; *HPRT1*: hypoxanthine phosphoribosyltransferase 1; *YWHAZ*: tyrosine 3-monooxygenase/tryptophane 5-monooxygenase activation protein zeta.

### 3.5. Specific intestinal bacteria

Adding specific fiber fractions derived from *Araceae* root and citrus to the piglet diet significantly reduced *E. coli* in the piglet caecum (Table 7). Furthermore, the fiber mixture in the test diet tended to increase *L. amylovorus* in the piglet small intestine, and significantly increased total bacteria count and Lachnospiraceae in the piglet colon.

**Table 7:** Specific bacteria in the small intestine (last 25%), caecum and colon of piglets at d14 post-weaning, when fed a control diet (Control; *n* = 10) or a diet containing 2 kg/ton specific fiber fractions derived from *Araceae* root and citrus (Test; *n* = 10) from d0 to d14 post-weaning.

**Specific intestinal bacteria (log10 gene copies/g of digesta)**	**Control**	**Test**	**SEM**	***P*** **-value**
**Small intestine**				
Total bacteria	11.13	11.30	0.22	0.42
Lactobacillus	10.36	10.57	0.37	0.59
*L. amylovorus*	8.51	9.70	0.44	0.07
*L. reuteri*	9.11	9.74	0.30	0.15
*L. johnsonii*	9.08	9.12	0.27	0.92
Streptococcus	9.22	8.97	0.37	0.64
Enterococcus	6.21	6.16	0.37	0.92
*E. coli*	8.15	7.21	0.70	0.12
**Caecum**				
Total bacteria	11.56	11.71	0.11	0.35
Lachnospiraceae	10.85	11.04	0.19	0.16
Ruminococcaceae	9.82	9.95	0.24	0.46
Bacteroides	7.63	7.44	0.25	0.60
Bifidobacterium	7.38	7.58	0.27	0.28
Clostridium	7.71	7.95	0.62	0.43
Lactobacillus	10.62	10.69	0.26	0.85
*E. coli*	8.91	7.72	0.36	0.03
**Colon**				
Total bacteria	12.14	12.54	0.37	0.05
Lachnospiraceae	11.31	11.63	0.10	0.03
Ruminococcaceae	10.40	10.51	0.54	0.64
Bacteroides	8.39	8.37	0.46	0.96
Bifidobacterium	7.85	8.14	0.27	0.23
Clostridium	8.26	8.35	0.64	0.81
Lactobacillus	10.98	10.82	0.60	0.83
*E. coli*	8.99	8.14	0.36	0.12

### 3.6. SCFA

Total SCFA were significantly higher in the piglet caecum and tended to be higher in the piglet small intestine for the piglets fed the diet containing specific fiber fractions derived from *Araceae* root and citrus compared to the control diet (Table 8). In the caecum, this was mainly caused by an increase in acetic (*P* = 0.007), propionic (*P* = 0.02) and butyric acid (*P* = 0.07).

**Table 8:** SCFA composition in the small intestine (last 25%), caecum and colon of piglets at d14 post-weaning, when fed a control diet (Control; *n* = 10) or a diet containing 2 kg/ton specific fiber fractions derived from *Araceae* root and citrus (Test; *n* = 10) from d0 to d14 post-weaning.

**SCFA (mmol/kg)**	**Control**	**Test**	**SEM**	***P*** **-value**
**Small intestine**				
Total SCFA	19.39	50.65	11.10	0.06
Acetic acid	6.03	12.63	5.50	0.37
Propionic acid	0.20	4.48	3.17	0.33
Butyric acid	0.13	2.49	1.74	0.32
Valeric acid	0.09	0.41	0.30	0.34
Lactic acid	12.80	30.45	8.16	0.15
Isobutyric acid	0.005	0.03	0.02	0.41
2-methylbutyric acid	0.08	0.08	0.08	0.99
Isovaleric acid	0.07	0.08	0.08	0.63
**Caecum**				
Total SCFA	83.94	129.86	12.60	0.01
Acetic acid	49.15	74.00	5.71	0.007
Propionic acid	19.68	30.98	3.68	0.02
Butyric acid	10.36	19.12	3.48	0.07
Valeric acid	1.54	2.78	0.71	0.23
Lactic acid	2.61	2.36	0.72	0.80
Isobutyric acid	0.15	0.20	0.05	0.50
2-methylbutyric acid	0.17	0.18	0.06	0.90
Isovaleric acid	0.27	0.26	0.04	0.86
**Colon**				
Total SCFA	91.32	104.69	19.30	0.16
Acetic acid	50.93	59.87	8.96	0.10
Propionic acid	21.02	23.50	5.06	0.33
Butyric acid	12.94	14.12	4.57	0.59
Valeric acid	2.29	2.10	0.57	0.58
Lactic acid	1.86	2.55	0.38	0.21
Isobutyric acid	0.92	0.99	0.13	0.70
2-methylbutyric acid	0.62	0.68	0.10	0.65
Isovaleric acid	0.74	0.87	0.11	0.42

### 3.7. Fecal biomarkers of intestinal inflammation

The MPO concentration tended to reduce in the feces of piglets fed the diet with specific fiber fractions derived from *Araceae* root and citrus (Table 9). No significant differences on PAP/REG3a and calprotectin concentrations were observed.

**Table 9:** Biomarkers of intestinal inflammation in the feces of piglets at d14 post-weaning, when fed a control diet (Control; *n* = 10) or a diet containing 2 kg/ton specific fiber fractions derived from *Araceae* root and citrus (Test; *n* = 10) from d0 to d14 post-weaning.

**Biomarkers^a^**	**Control**	**Test**	**SEM**	***P*** **-value**
PAP/REG3a (ng/fecal g)	18.3	16.8	3.2	0.64
MPO (ng/fecal g)	20.2	10.4	3.5	0.07
Calprotectin (ng/fecal g)	53.2	58.3	4.1	0.39

^a^MPO: Myeloperoxidase; PAP/REG3a: Pig Regenerating Islet Derived Protein 3 Alpha REG3a.

## 4. Discussion

Enterotoxigenic *E. coli* is an important cause of diarrhea in weaned piglets. A crucial initial step in the establishment of diarrhea, is the adherence of *E. coli* to the receptors present on the enterocytes of the small intestine. The inhibition of this bacterial adherence can be an effective way to prevent the initiation of the infection process. The adherence of bacteria to the intestinal mucosa might be inhibited by the use of ingredients that resemble the host receptors. These receptor-like or decoy ingredients can then bind pathogens, facilitating their excretion via the feces. As a result, *E. coli* concentrations in the ileum will reduce, leading to a decrease in the risk of adhesion and proliferation (24). It has been shown that dietary fibers can act as decoy receptors. Molist et al. (25) demonstrated that supplementation of 4% wheat bran in the diet of weaned piglets prevented *E. coli* adhesion to the ileal mucosa, reduced the *E. coli* population in the ileal mucosa, and decreased the severity of diarrhea. Mannose-containing receptor analogs were shown to be very suitable adhesion-inhibiting compounds (7, 8). In our study, it was hypothesized that the mannan rich *Araceae* root fiber fraction could prevent *E. coli* adhesion. Piglets fed the diet with specific fiber fractions derived from *Araceae* root and citrus tended to have lower *E. coli* colonization of the mucosal epithelium, which may indicate a lower adhesion of *E. coli* to the receptors of the small intestine. In support of this hypothesis for lower adhesion is the numerical decrease in the *E. coli* concentrations observed in the distal small intestine, as lower adhesion will lead to a lower replication of *E. coli*. Furthermore, the piglets fed the diet with specific fiber fractions derived from *Araceae* root and citrus tended to have a better fecal consistency score during the first 14 days post-weaning. Possibly, the lower adhesion and colonization may have reduced the production of enterotoxins, resulting in a better fecal consistency.

The reduced adhesion of *E. coli* to the intestinal wall was also reflected in the lower MPO concentration in the feces of piglets fed the diet with specific fiber fractions derived from *Araceae* root and citrus. When *E. coli* adheres to the epithelial cells and colonizes the small intestine, greater numbers of neutrophils are being attracted into the intestinal lumen (26). As MPO is a specific marker of neutrophil activity (27), the lower MPO concentration in the feces can be an indicator of lower *E. coli* adhesion and less intestinal inflammation.

Next to the *E. coli* adhesion, also the effect of the specific fiber fractions on immune responses were examined. Dietary fibers can affect immunity indirectly, via the production of SCFA or via changes in the gut microbiome. In our study, significant changes in specific intestinal bacteria and SCFA concentration were observed when specific fiber fractions from *Araceae* root and citrus were added to the piglet diet. Not only a decrease in *E. coli* was observed, but also *L. amylovorus* in the piglet small intestine tended to increase, and an increase in Lachnospiraceae in the piglet colon was detected. Lachnospiraceae are known as butyrate-producing bacteria (28). Butyrate concentration indeed tended to increase in our study, although this was only observed in the caecum and not in the colon. Possibly, other butyrate-producing bacteria that were not quantified in our study, could also have been increased by the addition of specific fiber fractions from *Araceae* root and citrus to the piglet diet and may have caused this tendency to increase cecal butyrate. Butyric acid is the preferred energy source for the colonocytes and is known to regulate cellular differentiation and proliferation within the intestinal epithelium, hence butyric acid helps to maintain a healthy epithelium and efficient nutrient uptake (29, 30). Therefore, positive effects on the histo-morphological indices such as villus/crypt ratio and villus surface area were expected, however these differences were not significant.

Dietary fibers can also have direct immunomodulatory effects, as specific fiber fractions can have the capacity to bind TLR (10, 12), and in this way may interact with e.g., LPS sensing. Normally, the stimulation of TLR4 by LPS activates cells through the NF-kB signaling pathway, which leads to a higher production of pro-inflammatory cytokines that are necessary to activate immune responses (4). Dietary fibers, such as pectins, can reduce inflammation, by prohibiting the binding of LPS to TLR4. This can be done via different mechanisms, e.g., pectins can bind to TLR4 and cause competitive inhibition with LPS (11), or pectins can bind to LPS and decrease its binding to TLR4 (14). Sahasrabudhe et al. (12) and Beukema et al. (13) demonstrated even a true blocking of TLR receptors by pectins. Although we did not find any direct immunomodulatory effects that could confirm the hypothesis that the specific fiber fractions from *Araceae* root and citrus could bind to the TLR receptors, we did observe some interesting numerical differences in our study which could point in this direction. A numerical decrease was observed in the gene expression of both NF-κB subunits *NF-*κ*B1* and *RELA*, which may indicate a lower stimulation of the NF-kB signaling pathway by the addition of the specific fiber fractions derived from *Araceae* root and citrus to the piglet diet. This also corresponds with the numerical decrease in the expression of the pro-inflammatory cytokines *IL1*β and *IL6*, and the numerical increase in the expression of the anti-inflammatory cytokine *IL10*.

In general, the outcome of this study showed only few significantly different results. A possible explanation could be the low diarrhea incidence in the control group, which could have been caused by various factors. Possibly, the infection pressure may have been too low during this study. An ETEC or LPS challenge trial in order to induce diarrhea or an inflammatory response could be a proper set up for future research. Next, the diet formulation of the control diet was maybe not challenging enough, e.g., due to the low crude protein level and the presence of gut health related feed additives, and this formulation could be adapted for a follow-up study. Furthermore, a genetic analysis to assess the ETEC susceptibility of the piglets was not performed in this study. Although the chance to find F4 or F18 *E. coli* genetically susceptible pigs in the Flemish pig population is 98% according to Nguyen et al. (1), it cannot be excluded that not all animals in this study were ETEC susceptible, or that an unbalance in the percentage of susceptible animals in the two dietary treatments could have occurred. The selection of piglets based on genetic markers associated with ETEC susceptibility should therefore definitely be included in a follow up ETEC challenge study (2). Finally, it cannot be excluded that a higher inclusion rate of the fiber mixture could have resulted in more significant differences.

To conclude, this study provided us with the indication that the addition of specific fiber fractions derived from *Araceae* root and citrus to the piglet diet after weaning can decrease *E. coli* colonization of the mucosal epithelium of the small intestine. Furthermore, it may reduce intestinal inflammation, possibly through both direct and indirect immunomodulatory mechanisms. However, to significantly proof this, a follow-up challenge study with confirmed ETEC susceptible piglets needs to be conducted.

## Data availability statement

The original contributions presented in the study are included in the article/supplementary material, further inquiries can be directed to the corresponding author.

## Ethics statement

Ethical review and approval was not required for the animal study because based on the European Directive 2010/63/EU on the protection of animals used for scientific purposes, no licensed ethical approval was required as this study did not involve procedures causing harm equivalent to, or higher than, that caused by the introduction of a needle in accordance with good veterinary practice, and because animals were killed solely for the use of their organs or tissues. Written informed consent was obtained from the owners for the participation of their animals in this study.

## Author contributions

ST, MD, JD, KL, JV, RD'I, and JM contributed to conception and design of the study. JD and JM conducted the research. ST and MD performed the statistical analysis. ST wrote the first draft of the manuscript. All authors contributed to manuscript revision and editing, read and approved the submitted version.
